# A Lecture upon the Discovery of the Circulation of the Blood, and the Theories of Generation

**Published:** 1857-04

**Authors:** Christopher Johnston

**Affiliations:** Lecturer, &c. Baltimore College of Dental Surgery.


AMERICAN JOURNAL

OF

### DENTAL SCIENCE.

Vol. VII. NEW SERIES—APRIL, 1857. No. 2.

### ORIGINAL COMMUNICATIONS.

### ARTICLE I.

*A Lecture upon the Discovery of the Circulation of the Blood, and the Theories of Generation.* By CHRISTOPHER JOHNSTON, M. D., Lecturer, &c. Baltimore College of Dental Surgery.

GENTLEMEN:

"What come ye out for to seek?" the agreeable or the true? and in return, will you, perhaps, ask me the question, What is it you purpose to teach us, captivating theory, entertaining history, or wonderful phenomenal reality?

And this have I to answer: We are all, indeed, fellow seekers after truth, and we ought to be equally in earnest in our search, equally single-minded in so noble a pursuit, and equally zealous in the attainment of our object. We strive to reach that which is like a fair city in some remote country, between which and us lie wastes and forests and morasses—there is spread before us an ocean of doubt and uncertainty, and the constellations above afford us no help, for they belong to an unfamiliar hemisphere. Some advance positively little, because the temptations which beset the way, divert the willing foot into profitless wanderings; some look back towards the starting point and remain fixed as the cubic crystals of Lot's wife, and some, full of hope, and love and trust, press evermore forward, and find in their nearer approach to the goal, the sweet satisfaction of duty performed.

I intend to deal mostly with facts, and particularly with those facts which stand in closest relation to the requirements of your daily studies—to demonstrate to you, as far as possible, *what is*, and not weary you with what is supposed to be—to show you how nature is to be detected in the midsts of what were once called her mysterious operations; and to weigh the value of what we may discover, and make the legitimate application of what we may positively ascertain.

You will, nevertheless, bear in mind, that physiology seldom rewards the most patient labor with the *wherefore*, while she allows the *manner* of functional action to be observed. There may be found in certain authors, the most delicate apprehension and the utmost exactness of description, while in the inferences drawn from what they have seen and recognized, there may be a constant misuse of the terms *how* and *because.* Thus we are informed that muscle contracts *because* its ultimate sarcodic elements change the relation of their diameters—that is, they become broader and shorter. But this alteration in figure obtains for the muscle itself, and we may easily suppose it for each of its contractile constituents. To arrive at a fair understanding of the matter, we must eliminate all but the muscular fibril; and, in spite of the answer that has been given, we are constrained to pursue the inquiry by demanding why do the affiliated sarcodic elements change the relation of their diameters?—in other words, why does a muscle contract?

Again, it is stated, that the yelk undergoes segmentation, *because* the ovum has received the fertilizing influence of the spermatic particles. The *fact* is asserted, but not the *reason* for it: and we may still ask, *why* does contact of spermatozoids and a mature ovum occasion segmentation of the vitellus? It were better to say that fission of the yelk is consequent upon contact of the spermatic fluid with the mature ovum.

The efficient cause of the phenomena of life, or indeed of things generally, is not better understood by the professor than by the student; by the natural philosopher than by the rustic. Shakspeare has keenly satirized physics or natural philosophy in "As you like it," when Touchstone asks Corin, "Hast any philosophy in thee, shepherd?"

Corin answers: "No more but that I know, the more one sickens the worse at ease he is! and that he that wants money, means and content, is without three good friends. That the property of rain is to wet, and fire to burn: that good pasture makes fat sheep; and that a great cause of the night is lack of the sun; that he that hath learned no wit by nature nor art, may complain of (the want of) good breeding, or comes of a very dull kindred."

"Such a one," rejoins Touchstone, with a quibble, "is a *natural* philosopher."

And farther, I think it not amiss to call to your mind the difference between consequences and coincidences—a distinction which it is indispensable to keep constantly in view while attempting to appreciate the true relation of facts made known to us by experiments of every kind, as well those of which we furnish the principal conditions, as those of which nature supplies all the conditions, and of which she vouchsafes a knowledge to the attentive observer. Thus, if we feed an animal—a carniverous animal—upon purely nitrogenous matter—supposing that this contains no saccharine compound in its substance or its juices—and we detect sugar in notable quantity in the hepatic tissue; it does not thence follow that the formation of sugar necessarily devolves upon the liver, either as a chief or as a secondary function. To estimate rightly the connection of *consequence* between the absence of sugar in the stomach and the presence of that particular hydro-carbon in the liver, we must convince ourselves that sugar exists neither in the intestines nor in the portal veins—that the reagents which we employ are proper, pure and certain in their action; and that

*there*, where we fail to detect the object of our search, nothing is locally exercising its influence to screen the saccharine matter from the action of a test which is efficient elsewhere.

What is constant is essential—if, then, we find no sugar in the food, the stomach, the intestines or the portal veins; if we find none in the spleen, and, at the same time, if we meet with it as a constant constituent of the blood issuing out of the liver through the hepatic veins, we *are* justified in concluding that the latter organ presides over the elaboration of sugar, while in the absence of these correlative facts, we are constrained to regard the non-existence of sugar in the food and stomach, and its existence in the liver as independent of each other. In the first instance we have a *consequence;* in the second, a coincidence. Now, this sort of reasoning, so just and commendable, must find its application, not in physiology only, but also in every other department of medical science; and while it narrows the domain of unreal knowledge, it gives greater exactness to inquiry and precision to the expression of results. Guided by it, voluminous writings have been sifted and the few grains of wheat have been rescued from the chaff, and treasured up with other solid acquisitions—*dicta* of the most reserved authorities have been reduced in estimation to opinions—*laws* have been abrogated, which were in fact suppositions to make way for real generalizations of facts, and an honest confession of ignorance is frequently made where there had been else a bold but groundless assumption.

You must not, however, imagine that the great truths of our art were ever arrived at by logic, or that the fathers of medicine and the collateral sciences sought to *substitute* their ideas for the teachings of nature. On the contrary they used faithfully every atom of knowledge they possessed, and *then* eked out their story with the probabilities which they admitted. So exact now, in general, their observance of phenomena that their accounts of them are current at the present day; and the laws they sought to establish, and which have stood the test of time, are precisely those which were based upon the *greatest number* of observations. Still, it cannot be denied that the latter fell into unwarrantable disuse; and the tenets of Galen held sway for centuries even in opposition to the truths elicited from nature by experimental philosophy.

While speaking of the *ancients*, I would beg leave to remark that their greatness did not depend solely upon the fact that the field of knowledge was free and uncultivated—that to be original required only to walk observingly abroad and cull the fruits which opportunity shook from every tree. They were great not only in the *matter* which they laboriously accumulated, but also in the *manner* in which they used it, and such *moderns* as imitated their industry and garnered what *they* reaped with equal care, have not been denied as green a wreath as any which bound the brows of men of remotest ages. Nevertheless, in the award, while the amount of labor accomplished decided in general terms the quality of the prize, there must be taken into consideration the circumstance that the beacons set up by the older men shed an extremely advantageous light upon the part of all who followed them.

Notwithstanding that the justly renowned Ambrose Paré, appeared in the intellectual arena about nineteen hundred years after Aristotle, it may be plainly seen that the novelty of nature, (if I may so express it,) had nothing to do with the quantitative or qualitative labor accomplished by these men, if we compare the "History of Animals," by Aristotle, with the "Treatise upon Monsters," by Paré. Of the latter we may say, that it is singularly curious, but is unreliable, because its statements are based upon impressions, popular belief, and reports of gentlemen in the marine service. With regard to the former we may not do better than quote the apposite remark of Buffon: "The History of Animals of Aristotle," says Mons. de Buffon, "is still, perhaps, the best of its kind that we have even at the present day. He had probably a better acquaintance with animals and under a more general aspect than they are now known to us, although the moderns have added their discoveries to those of the ancients. I am of opinion that there are few works upon Natural History, which can be placed above those of Aristotle and Pliny."

In any similar point, the question at issue is *not where* the work was done, but *how* the work was done.

I take leave of these generalities, gentlemen, to approach the subject-matter of those things which are to occupy our attention—and I know of no better or more profitable manner of employing this hour than by endeavoring to entertain you with the *history* of a few of the discoveries which shed lustre upon some of the greatest names in medical science, and formed the basis of modern physiology.

"The discovery of the circulation of the blood," observes that distinguished savan Flourens, "does not belong, nor can hardly, in fact, belong to one man nor even to a single epoch. Old errors had to be destroyed, and a truth substituted for each of them. The whole thing, therefore, was done successively, slowly, little by little."

To follow this discovery in its various periods of origin, advance, neglect and reiteration, is not a little interesting and instructive; for these are marked with the footprints of men of the greatest genius.

Hippocrates, who lived 460 B. C., appears to have been the first who knew or understood, however vaguely, the circulation of the blood. It is impossible that it could be otherwise adjudged—for in spite of the confusion which some of his impressions might occasion, there are not wanting passages in his works which are explicit to the last degree upon this point.

He says, in the very words I employ, that the heart is the source of the arteries which conduct the blood, the spirit and the heat into every part.

That the veins are all branches of a single trunk, and communicate with each other and flow into each other. They are distributed throughout the body, and are, as well as the heart, unceasingly in motion—they bear the spirit, flux and motion everywhere; and he compares the course of rivers, which return to their source by extraordinary channels, to the complete course of the blood.

If this be not sufficient to establish the proposition, I will quote the master more at length: "I confess," says he, "that I

do not know where the great vein has its beginning, or where it ends—but obstruction in the veins, whereby the passages are intercepted, occasions apoplexy or other similar diseases; and he counsels blood-letting in order to procure or bring about a free movement of the blood and spirits: and finally, I adduce the crowning remark; that as the thread of the knitter or weaver, being knit into a globular figure, ends at its beginning; so in the body there is a circuit—*where* it begins, *there* also does it terminate.

I am at a loss to comprehend the objections which have been urged against such proofs as these, as I am astonished that the truths contained in the writings of Hippocrates, although mingled with error, should have passed unnoticed or neglected for centuries after he had ceased to exist.

Before the time of Galen, (A. D. 193,) however, the apparatus of heart, veins and arteries was partially understood; but the inter-ventricular septum was thought to be cribriform; the arteries were supposed to contain *air*, which, penetrating into them through the aspera-arteria or trachea and the venous-artery or pulmonary vein, tempered the body; and the veins were believed to contain and distribute blood.

Galen made a great step forwards, a step which brought the discovery of the circulation of the blood almost within his grasp; he established the fact that the arteries also, were blood vessels, thereby setting aside the ancient division of systems into the *arterial* or *aërial*, and the *venous* or *sanguiferous*. He says, "whenever an artery is wounded we see blood issue out of it—wherefore one of two things must occur, either that blood is contained in the arteries, or that it flows into them from elsewhere. But if the latter were the case, it is evident that since the arteries should properly contain nothing but air, the air ought to escape before the blood. Now since this does not take place the arteries contain blood only."

Again: Galen practices another experiment. He encloses a portion of an artery between two ligatures; he opens the intercepted portion and finds blood and nothing else—wherefore once more the arteries contain blood only.

But what, cried out his opposers, if the arteries contain blood, how does the inspired air pass into every part of the body? It does not so enter in, answered Galen, but is expelled again. It acts by reason of its temperature and not by its substance—it cools the blood; and this is the use of respiration.*

What was now wanting to bring to light the circulation of the blood, which in some sort Hippocrates had proclaimed, and which the continual flow of the blood would almost seem to call for? It was the belief that the inter-ventricular septum was perforated in order to permit the passage of a part of the spirit generated in the left ventricle into the right—and the fact, that there are two distinct sorts of blood, the *spirituous blood*, destined for the more delicate organs, and the *venous blood* appropriated for the nutrition of the grosser organs. You will bear in mind that Galen recognized *three* spirits—1st the natural, 2d the vital, and 3d the animal. The *first* had its seat in the liver, the *second* in the heart, and the *third* in the brain: and furthermore the *animal spirit* arises out of the *vital.*

The vital spirit, says Galen, in his treatise, "On the Use of the Parts of the Body," is the exhalation of the blood. Wherefore the *vital spirit* originates in the vapor of the blood in the heart, especially in the *left ventricle;* and from the *vital spirit* infused into the arteries, the retiform plexus and the ventricles, arises the *animal spirit.* "The *animal spirit,* the spirit born in the brain, is the most noble and exquisite part of the human body, it is the proper substance of the soul, or is, at least, its first instrument. Reason, which is Man, has its seat in the brain; whence the ingenious fiction of the birth of the Minerva from the brain of Jupiter."†

It is not to be wondered at, that if so great a master suffered his ideas concerning the circulation to rest here, the Galenists, his disciples, allowed no innovation. How could it be otherwise, when for centuries after his death, observes Beck in the History of Medicine, his doctrines and tenets were regarded almost in the light of oracles, which few persons had the right to oppose; and all improvements in medicine existing or con-

*** Flourens, de la Circulation du Sang.** **† Flourens, (ant. cit.)**

templated were nothing more than illustrations of his doctrines or commentaries on his writings. In numberless instances it was deemed a sufficient argument, not merely against an hypothesis, but even against an alleged matter of fact, that is, was contrary to the opinion of Galen: and it may be stated without exaggeration, that the authority of Galen alone was estimated at a higher rate than that of all the medical writers who flourished during a period of more than twelve centuries." Even Vesalius taught that "the greater portion of the blood passed through the pores of the inter-ventricular septum from the left ventricle into the right;" and *this* in spite of his own conviction; for he afterwards declares that he did so simply in conformity with the views of Galen.

The sixteenth century, gentlemen, was a noble era in art and science. It was the age of Raphael, Michael Angelo and Leonardo da Vinci—of Titian and Guido and Corregio—Ariosto and Tasso sang with inspired lips; and Shakspeare, Prometheus like, stole fire from heaven to vivify his creations. Artillery was first used, and modern warfare had thus its beginning, under Francis I, at the beginning of this century; and the silken ligature, invented by the renowned Ambrose Paré towards its conclusion, first arrested arterial hemorrhage. The microscope was sketched out just before the commencement of a new epoch—while in the very middle of the sixteenth century the *circulation of the blood* was formally announced to the world.

Michael Servetus, to whom the honor of the discovery is due, was a native of Arragon, in Spain, and the son of a notary. He studied *law* at Toulouse; but manifesting a stronger affection for *medicine,* he studied at Paris, took the doctor's degree, and subsequently established himself as a practitioner of medicine in Dauphiny. Not content to seek celebrity in medical pursuits, and eager to publish his Arian religious opinions, he sent three "questions" to Calvin, (who may be said to have ruled in Geneva,) "on the divinity of Christ," "on regeneration," and "on the necessity of baptism." His ardent temper changed the peaceful nature of the controversy he had begun into a bitter correspondence; and Calvin, became in consequence his unrelenting foe.

In 1553 Servetus published a book embracing most of his previous writings, and which have this title "Christianismi Restitutio." The revival of Christianity; whereupon Calvin caused him to be accused as a dangerous man, and his imprisonment followed. Escaped from prison he attempted to pass through Geneva in disguise, but was seized by the authorities as an impious heretic—the magistrates, urged especially by Calvin, condemned their prisoner as guilty of forty heretical errors; and upon his refusing to renounce them, was burnt at the stake a few months after the publication of his book, on the 27th October, 1553, and died after two hours torture.

The only two copies extant of this remarkable work belong to the imperial libraries of Paris and Vienna, and that of Paris is the very same which was used by Colladon, one of the accussers instigated by the implacable Calvin against Servetus. Colladon underlined the propositions which served as the basis of the accusation—and this volume, somewhat scorched by fire, was only snatched from the pile, upon which the author and his books were to be burned, when the flames had already began to ascend.

It is not a little curious that the pulmonary circulation should find a place in theological writings, but this is the occasion of its mention.

The point to be demonstrated was, the soul of all flesh is in the blood—the blood is the soul itself—the scriptures declare *anima est in sanguine; anima ipsa est sanguis.*

"Since, then, the soul is in the blood, says Servetus, to know how the soul is formed, it is necessary to know how the blood is formed; to know how the blood is formed the manner of its motion must be understood; and thus while treating of the revival of christianity is he led to the formation of the soul, from the formation of the soul to that of the blood, from the formation and motion of the blood to the *pulmonary circulation.*"* He then goes on to advance a complete explanation of the soul and the spirits; and he says, "I will unite with it a divine philos-

*Flourens.

ophy which you will easily comprehend, however little you may be skilled in anatomy." His theory of the spirits was the same as that of Galen.

I will now further quote the author that you may see with what precision he speaks of the circulation in the lungs. He treats of the course of the blood. "But," says Servetus, "the communication of the two ventricles does not take place through the walls of the heart, as is commonly believed, but by a wonderful artifice, the blood passes from the right ventricle by a long route through the lungs, where it is agitated, prepared, when it becomes yellow, and passes from the *arterial vein* (pulmonary artery) into the *venous artery* (pulmonary vein.) * * And this is confirmed by the calibre of the arterial vein, which would not be so large, nor would it convey so great a volume of blood to the lung, if the nutrition of the organ were the only object: the more especially," continues the author, "since in the embryo the lung derives its nourishment certainly elsewhere, since the blood does not reach it by this channel. And further on; "In the same manner that in the liver the passage of blood takes place from the portal vein into the vena cava, so also in the lung is effected the transfusion of blood from the arterial vein into the venous artery."

Here have we not only the passage of the blood, the manner of the transit, but also the precise spot where sanguification is effected—and expressed, too, with a clearness not surpassed in modern works. Why did he stop here, why did he not extend his reasoning to the body at large when he had already overcome every difficulty in two of the most important organs of the economy?

The merit of rendering the discovery complete was reserved for another, and this was Andreas Cesalpinus, who likewise spoke of the valves at the openings of the great vessels, and described the circulation of the sap in plants and their organs of fructification.

In 1571, just seven years before Harvey, the great Harvey, was born, Cesalpin gave to the world his book, entitled, "De Quæstionibus Peripatecis," and a few years later his "De Quæstionibus Medicis." "It is a very singular thing," says he, "that the veins swell *below* the ligature and not above it. Those who bleed sick persons are familiar with this experiment; they always place the ligature *above* the point at which they wish to draw blood and not *below* it: the which ought to be just the reverse if the motion of the blood were (in these vessels) from the heart towards the parts."

Again, he says in another place, "the apertures in the heart are so disposed by nature as that the intromission (of blood) is made *from* the vena cava *into* the right ventricle of the heart, whence its exit is into the lung: it has, furthermore, another ingress into the left ventricle of the heart from the lung; out of which at length it makes its exit into the aorta—and *certain membranes* are adjusted around the mouths of the vessels so as to prevent retrocession. Thus there is a certain perpetual motion from the vena cava through the heart and lungs into the aorta," and now do we meet, for the first time, with the distinct expression "*circulation of the blood.*"

If you have followed me throughout the whole of the exposition I have given of the present subject, you will have perceived that the foundation of this great discovery was prepared by Hippocrates, but, as any other foundation, was not a conspicuous object—that the views of Galen were more comprehensive on this point, yet were still clouded in a mysterious atmosphere of coctions and spirits—that Servetus, without altogether ridding himself of the confusing notions of spirits, positively described and published the pulmonary and hepatic circulations—and lastly that Cesalpin finished the work, proved the course of the blood in the veins to be towards the heart, asserted the general circulation, using the very word *circulation*, and spoke directly and unequivocally of the valves at the orifices of the great vessels of the heart as well as of their use.

I might stop here, but you would wonder why I pass over in silence the generally admitted claims of *William Harvey.* I have no intention, however, of withholding from Harvey the meed of great praise which is his due; but I set myself the task of reviewing the authors whom I have mentioned, in strictly chronological order, and consequently *he* must appear *last* upon the scene.

Harvey was born April 2d, 1578. At the age of nineteen (19) he travelled through France and Germany; and, attracted by the reputation of the celebrated Fabricius di Acquapendente, he proceeded to Padua, and applied himself to the study of medicine under that great master. He returned to London with all the information upon the subject of the circulation which was then to be gathered—in 1615 he was made lecturer on anatomy and surgery—in 1616 he laid open the discovery of the circulation of the blood in his lectures, and in 1628 he published his "Exercitatio Anatomica de Motu Cordis et Sanguinis," his masterpiece, and dedicated the work to Charles I, after having verified the statements it contained by many years' experiments.

Now, after what has been stated, *wherein* lies the great merit of the English philosopher? and *how* has his connection with the greatest discovery in physiology redounded to his almost exclusive credit?

I will reply to these interrogatories and terminate this chapter by quoting the appropriate remarks of Flourens upon this very point; repeating what I have already observed, "that the circulation of the blood belongs neither to a single man nor a single epoch: This great discovery was made little by little, part by part: more than twenty anatomists coöperated in it."

"Harvey," says the distinguished Flourens, "demonstrates the circulation of the blood; but he comes from Padua, where he had for his master Fabricius di Aquapendente who discovered the valves in the veins; but in this same University of Padua, where the first germ of all the ideas of Harvey had its birth, Realdo Columbo had recently held a professional chair, he who independently, but six years subsequently to Servetus, had discovered the pulmonary circulation; but Padua is not distant from Pisa, where Cesalpin, in a burst of genius, more than suspected the pulmonary circulation, and in another inspiration perceived the general circulation."

I may add, in this place, that Padua is much less remote from Venice, where Cesalpin published his remarkable work from which my citations were made. "In this discovery," continues Flourens, "the difficult point was to connect the divers parts, and, if I may so express myself, the various pieces, successively brought to view, into a unity—the difficult point was to seize the comprehensive whole of the phenomenon, of the mechanism; and it is because Harvey is the first who did so grasp, clearly and completely, the phenomenon in its entireness, that the great glory has been awarded to *him*."

I now propose to occupy your attention with a subject possessing no less interest than that to which you have so kindly listened; but which from its mysterious nature, and from the limited means of observation of the earlier investigators, was, if I dare to say it, the romance of physiology. The wisest sought to penetrate its mystery; the presumptuous, hazarded their speculations with the more confidence, since there were none who could speak with more than speculative probability; and it was not until nearly seventeen hundred years of our era had passed away, that truth and certainty shone forth after a long night of theory and uncertainty. I have reference to the *origin of a new being.*

There is, perhaps, nothing which can awaken so high a degree of admiration in the human breast as the starting into life of a new individual—the extension of the existence of other individuals whom dissolution must inevitably overtake—and *which vitality* is an endowment of trust not intended exclusively for the use of its present possessor. Whilom a mere speck in the most hidden parts of the adult organism, it has passed through numberless metamorphoses, each one of which corresponds to an especial condition. In the secret laboratory of nature its various elements are formed, and completed, and fashioned to the likeness of the parents from which it springs—the spiritual part affects a rudimentary state in unison with its really rudimentary corporeal agent; and what is very wonderful, organs which minister to the present wants of the immature creature are holding in reserve capacity for actions which an instant may call into requisition.

And that instant arrives. The wrappings which formed its bonds of dependency are cast off one after another; the forces brought so harmoniously into play are urging it forward towards the field of its definitive independence; and when, at length, the separation from all attachments is accomplished, the organs of its separate existence begin to function, and its new career—its relation with the great world—has begun.

It is not, therefore, surprising that the genesis of living organisms, and particularly of our own species, should have engaged the attention its interest would justify. In our own times, with the superior advantages we enjoy, and with the great amount of positive information we possess, the subject has lost none of its charms. But our inquiries are directed in a different spirit from those of men of the olden time; and the assistance which our vision has received from the microscope has made us tread firmly where before the footing was yielding and insecure.

To appreciate our present position, it cannot be otherwise than serviceable for us to cast a retrospective look upon the position of those who have preceded us; to view the matter with an eye unassisted save by the noblest attribute of the mind, and to watch the workings of genius, which, before the advent of the microscope, confident of its acquisitions, sighed for new worlds to conquer.

It is impossible that the existence of *two sexes* in all known animals should have been disregarded by the earliest natural historians in their contemplations upon the subject of generation. All the fables of mythology allude, in distinct terms, to the concurrence of male and female in the generative act. Moses records that "on the sixth day God made the beast of the earth after his kind, and the cattle after their kind, and every thing that creepeth upon the earth after his kind—and created man in his own image—male and female created he them:" and the *necessity* for the union of the sexes was apparent in the every day experience that an isolated animal, male or female, was insufficient for obeying the command, "Be fruitful and multiply." The oldest idea of procreation is consequently the most natural, to wit: that it results from the concurrence or mixture of the sexes. But the notion once conceived, started the question, "How is this accomplished?" An inquiry involving, obviously, all the difficulties of the case.

1st. Two opinions seem to have divided antiquity in solving this question; 1st, on the one hand it was maintained that all animals form and arise out of an egg or ovum, and

2d. That they originate under the form and figure of little worms.

The first dogma, which may really be viewed as a discovery, was expressed and supported by Hippocrates especially, and by many others, among whom may be enumerated, Aristotle, and also Macrobius, who states definitely, that in all kinds of animals which copulate, an ovum is the first principle or element of their generation.

But Hippocrates, apparently not satisfied with advancing the ovular theory of reproduction, enters into certain speculations which form its basis of Leibnitz's arguments in modern times, concerning what is known of the theory of the "pre-existence of the germ."

The father of medicine, speaking of the formation of the child, describes a fetus of six days; he compares it to a crude egg of which the shell had been removed, which was globular and reddish, and in which there was a very transparent fluid; in another place he says, that nature is evermore the same, that she acts in a uniform manner with respect to the generation of men, of plants and of everything which has birth. And again, he advances, "that nothing new is produced—nothing is born which did not before exist: that everything grows or increases as much as possible, from the highest to the lowest degree—that the greatest grows out of the smallest—that all parts are developed equally, and not one before the other; but that the greater, by reason of their nature, *appear* at an earlier moment than the smaller, although they were not earlier begotten. And finally, he affirms that the birth of animals is nothing more than an accretion or development which conducts from darkness into the light."

More than two thousand years after these views emanated from the illustrious physician of Cos, they found a partizan in Leibnitz, and enjoyed a transient popularity under the authority, Bonnetus, Haller and Spallanzani.

Aristotle expresses himself more clearly upon the nature of generation; and in his History of Animals, he uses, without paraphrasis, the term *ovum.*

Animals, says he, are perpetuated, either in a living animal, an ovum, or a worm. We call *that* ovum, (when we speak of a perfect production of this kind,) whence comes an animal, which, formed in the beginning out of a portion of the egg, uses up the excess for its nourishment. The ovum has either a hard covering, and contents of two colors, as that of birds; or else it has a delicate envelop and contents of one color. But, observes the author in the same place, when we distinguish animals according as they produce a living animal, an ovum, or a worm, we speak of that which is made manifest *exteriorly* in in a complete production.

The *vermicular theory* is of almost as early a date as the *ovular*, but the latter, after indicating the form assumed by the germ in its first stages, leaves the mind quite at fault in regard to the part which the sexes take respectively in its formation.

Democritus, a cotemporary of Hippocrates, taught that men have their beginning by starting into existence under the form of little worms; and Aristotle, who succeeded him by about one hundred years, comprises the worm in his category of the first fruits of a perfect generation. But, as we have already seen, the Stagyrite explains himself fully upon this point, assuring us that the forms of which he speaks are but the outward manifestation of what takes place in divers animals.

Again, Plato, speaking of generation in man, compares the uterus to a fertile field, in which the seed or semen produces the fætus just as a tree produces its fruit; and continues the remark by observing, "that the animacules thus formed, and which here receive their growth, are at first imperceptible on account of their extreme minuteness, but little by little they appropriate the nutriment prepared for them in the uterus, and at length come to light in a state of generation."

And finally, a commentator of Seneca advances positively, that this philosopher must have had a very exact acquaintance with the system of generation by animalcules, since he teaches that the form of the man to be born is already comprised in the seed of man; and that all the members of the body are as it were, concentrated and shrunken into a small space.

It must be confessed that with whatever vagueness of expression it is set forth, the ovular theory commands our admiration and its first promulgation certainly merits the honor of a discovery, the truth of which is verified by all the researches of our own day; it reposes upon observation, the real source of knowledge, whereas the vermicular theory is vagueness itself, rests mostly upon speculation, and can, with no great difficulty, be made to sink into the former; nevertheless, you will presently perceive that this system was revived in a more definite form in later times, but that it met with the same fate as its predecessor.

Without making further citation, I beg leave to present to you a synopsis of doctrines of generation; reminding you, however, that while I revert to names which have already been mentioned, it will be merely to give an extension of their views, and not to repeat what has already been advanced.

Hippocrates admitted the existence of two spermatic fluids, each formed by an assemblage of molecules from every part and portion of the producing organism.

Upon the mingling of these fluids depended impregnation; and, according to the *equality* of the mixture, the resemblance in form and feature to both parents, or to the disproportion, (or preponderance,) the greater resemblance to one parent and *the sex.* But in the latter case he thought to avoid a difficulty by creating two additional fluids destined especially to the endowment of sexuality.

According to him, also, the conveyance of the molecules may find proof in the universally diffused sensations produced by sexual union, during which the particles are invited to congregate; in the consequent relaxation determined by their separation; and in the perpetuation of certain deformities, such as lameness.

Aristotle adopted and professed the same opinion, with this difference, however, that lameness is not necessarily transmitted from parent to child.

Galen principally surpassed his predecessors in knowledge of anatomy. He termed the ovaries the *female testicles*, a name which they bore for many centuries. The Graafian vesicles were for him reservoirs of the female spermatic liquor ; and its conduits were what we now call "Fallopian tubes," which he perfectly understood and examined with a probe. But their discovery is referred to Fallopius of Modena, (ob. 1562,) nevertheless, the following passage in reference to this point may be found in the work of Ruffius Ephesius, De partibus Corporis Humani ; and it is highly probable that the ancients were acquainted with them. "Herophilus," says he, "believed that women have no varicose epididymes ; but we have found, in examining the uterus of an animal, certain vessels which arise in the testicles, and which being folded hither and thither under the form of varices, terminate by one of their extremities in the cavity of the womb. * * * And it is believed that these are certainly seminal vessels of the nature of those called varicose, (or epididymes.)

Fabricius di Aquapendente, in the 16th century, was the first among the moderns who submitted the opinions of ancient philosophers to the test of direct observation, or in fact, to introduce the positive method.

Profiting by his instructions and example, the great Harvey, his disciple, performed *his* experiments, and made *his* observations upon generation in the presence of his king, Charles I. In spite of the objections of the latter, Harvey thought his proofs positive that the uterus is principally concerned in this process, and without its apparatus and function, we may hope in vain for conception. But forasmuch as it is certain that the sperm of the male parent does not reach the cavity of the uterus, it cannot remain there ; now it brings about fecundation, not because it touches and operates upon, (the uterus,) but because it has touched it ; in fact, a woman, after spermatic contact in coitû, may become pregnant, precisely as iron, which, by the magnetic touch, becomes endowed with power, and attracts other bits of iron unto itself.

He left it to be inferred that the ovaries are not essential, or rather superfluous, and finding in the mucous membrane of the uterus of some animals a peculiar corrugated arrangement resembling convolutions, he fancied that the uterus conceived a new being as the brain conceives an idea.

It is past all presumption that Harvey mistook the *place of origin* of the *germ*; nevertheless, his great mind, reducing to harmony the speculations of his predecessors and his own observations, entitled that memorable dictum, "Ex ovo omnia."

This was in 1651, the date of his book upon the generation of animals.

It is not surprising that such a man as Harvey should have arrived at this generalization, for in his time everything tended to give strength to the ovular theory.

From the earliest ages there existed a disposition to associate the egg of birds and reptiles with the fruit of conception in animals, and this with the seed and germs of plants. Empĕdōcles (A. C. 444) was of opinion that all that is born has its origin in a seed which he compares to an *ovum*; and herein is truly the grand idea of Harvey. Aristotle went a step further, and announced the *sexuality* of plants, described the difference between trees of different sex, remarked that the fruits of trees did not ripen unless they had previously been fecundated by the dust (or pollen) of the male flowers; and acknowledged the justness of the suggestion of Empĕdōcles, that plants are oviparous, for, says he, the *ovum* is the result of generation.

The poet Claudian, (A. D. 365,) in a burst of enthusiasm upon the "Force of Love," thus expresses himself: "The delicate branches live only for Venus, and the fortunate trees pass their time in an interchange of love; the palm tree, full of caresses, aspires to mutual embraces with the palm, and the elder, the plantain and the poplar, continually breathe out their endearments in sighs. "I leave," says he, "this poetical style to pass to the testimony of naturalists, who teach the sexual system of plants in a very unequivocal manner."

And finally I will adduce the authority of Pliny upon this point. In his Natural History, he compares the conjunction of plants with the copulation of animals; and says that in order to bear fruit it is only necessary for the females to receive the aspersion of the dust or down of the male flowers.

From what has preceded you must perceive that the discovery of the ovular theory is due to Empedocles and Hippocrates; that various disjuncta membra were scattered throughout the writings of the ancients: and that Harvey, with the true spirit of a philosopher, weighed all the opinions of others, for he was well acquainted with them and quotes their authors; instituted a long series of observations, and arrived at an expression of truth which is almost universal.

After Harvey, Steno prosecuted the study of generation, and revived the notion that the ovaries are the testicles of the female—whereupon the most lively discussions arose as whether Harvey's views should obtain, or whether the new being did not result from the admixture of the male and female procreative liquors in the uterus, the latter fluid reaching the womb either by the Fallopian tubes or by certain mysterious passages. This last doctrine had its supporters even at the end of the last century.

At this juncture appeared the little treatise of Regner de Graaf upon the Parts, male and female, concerned in Generation.

He had studied "the testicles of the female" as they were then called, and had found what he supposed to be *ova*, under the form of vesicles imbedded in their substance; but what attracted his particular attention was the fact the ova he detected in the *Fallopian tubes* and in the *uterus* were of much smaller dimensions than those he was accustomed to observe at their point of origin. Accordingly he pursued his investigations, incited thereto by his perplexity, and had the extreme gratification to discover the *true ovum* contained *in* the vesicles of the ovary which now bear his name.

*Harvey* defines the *ovum* to be "a something conceived, proceeding from the male and female, endowed equally by both, and from which unity there arises one animal."

De Graaf describes the *ovum* as a transparent vesicle contained in a capsule or vesicle in the ovary, from which it is liberated by the action of the spermatic fluid and fecundated—the male semen passing to the ovary through the Fallopian tube.

From this point he follows the ovum in its descent into the uterus and there studies its development, and casting a retrospective glance towards the ovary becomes acquainted with the formation of the *corpus luteum.*

These discoveries of De Graaf were a triumph to the ovarists: they spread with great rapidity, and were vaunted everywhere to the annoyance and discomfiture of their opponents. Much warmth grew out of the discussion—when, to oppose the extravagancies of the ovarists, Franciscus Plaatade, of Montpellier, in France, a hitherto unknown person, while travelling in Holland, revived the vermicular theory of the ancients, supporting his views by the discovery he pretended to have made by means of a powerful microscope of exceedingly numerous, active, subtle animalcules that swim in the human seminal fluid. His wonderful revelation began to gain credence and his partizans to grow confident, when to their disappointment he confessed that he had invented the deception simply for his amusement. But their mortification was of short duration, for one Ham, of Leyden, a student of medicine, while examining the spermatic fluid of the testicle under the microscope, really made the discovery of the spermatozoids—he recognized their rounded head or body and their filiform tail, and witnessed the extraordinary vivacity of their movements. He eagerly communicated his good fortune to Leeuwenhoek, his master, who was equally astonished and delighted.

The discovery of the ovum was quickly swallowed up in the new discovery, the novelty was bruited far and wide; and the importance of the ovum was almost lost sight of.

Leeuwenhoek (in 1722) pursued the study of these singular particles, proclaimed their animality, and considered them as homunculi, little men and women, wanting but a nidus, and this was the ovum, for their development. Seizing upon the idea of the pre-existence of the germ he applied it to the spermatozoid, he represented certain ones with the figure of an ass, and others in man's likeness. He made the homunculus to enter the ovule by his head through an accidental or a normal valvular opening, and supposed that while the *body* developed itself *within*, the *tail* engrafted itself *without*, and by metamorphosis became the placenta.

All this is strange enough, gentlemen, but still more remarkable are the refinements he made with regard to the *homunculi.* As animalcules the spermatic particles should have a complete organization. Leeuwenhoek endeavored to prove the existence of alimentary organs; then to establish their sexuality, and then as a natural consequence, supposed a miniature congress of the sexes. Next he delineated the envelops of the male and of the female; and finally assumed these envelops of the zoosperms to be the fætal envelops, and the placenta, (as we have already said,) the development of the caudal extremity.

However attractive and complete this theory may appear at first sight, it is evident the question at issue, namely, the generation of a new being, remained precisely where it was in the beginning, that is to say with the exception of the truth of the existence of the spermatozoids, for if these be animalcules and if they copulate, how then is the *homunculus itself produced.* The science of our day only has measurably removed the veil of mystery which hung over conception, and defined the true field of action of the moving particles of the sperm.

I trust, gentlemen, that you will bear with me patiently on this occasion, for I doubt if I shall again seek to enter into similar details or to engage your attention with *abstractions*, which, having no importance in practice, are of value only in their connection with the history of science. But, as I have before remarked, it is necessary for us to know the state of knowledge in times long since passed, and to follow, in a few prominent instances, the development of truth from its nebulous origin to its condition of evident and conspicuous strength. With regard to generation, I have led you through the mazes of hypothesis which characterized the ages preceding Fabricius di Acquapendente, Harvey and De Graaf—it now remains for me, before closing this Lecture, to make some remarks concerning the *positive period*, and of certain *theories*, interwoven with each other, which appear to derive their importance from a basis of real and alleged observation.

But even here you will be surprised to find that although one of these theories received its baptism in modern times, its nature was understood and proclaimed before the advent of our Lord; and that the other, which started into life fully formed in antiquity, is now tottering to decay under its very *age* and infirmities. These the moderns have renewed, revived or restored; but let us confer the palm upon the true originators for whatever merit should accrue from the inventions.

"It appears to me," says a learned member of the Royal Society of London, M. Dutens, "that the restorer of the *system* of some great man, of which we get only a glimpse from a few fragments of his writings, may be justly compared to a skillful sculptor, who finding a broken bust of Phidias, or of any other famous artist of antiquity, is able, by the help of his genius and his acquirements in his art, to estimate precisely all the relations of the members among themselves which belonged to this bust; to determine their precise proportions to the broken bust, to work them out, to join them, and out of them form a statue as perfect as apparently *that* was, of which this bust constituted the principal part. The merit of such a modern artist would deserve, doubtless, very great praise; but the glory of the ancient artist would ever be above his own, because it must be admitted that the ideas of the proportions of the added members would be derived from the suggestions of the broken bust."

The first of these *theories* are the theory encasement, or pre-existence of the germ, and its adverse theory of epigenesis; the former most distinctly sketched by Hippocrates in that remarkable passage I have quoted, and the latter embodied in the writings of many of the authors to whom I have referred. Leibnitz, and especially Bonnetus, revived the former notion, and were sustained in their views by Spallanzani and Buffon in the latter part of his career.

In a letter from Bonnetus to Spallanzani, dated Genthod, Nov. 1790, after the usual salutation he says: "You always know how to interrogate nature as she likes to be questioned. I meant to say that you are her spoiled child. But if we should never succeed in seeing distinctly the germ of the greater quadrupeds and of birds before fecundation, we would not for all that be less good logicians in presuming that this germ is pre-existent before fecundation, or in other words, that its formation is not due to the concurrence of male and female, and that it dates *à primordio.*"

In another place he says, "that 'encasement of germs' is an improper word; for the germs are not little boxes contained the one within the other; they were integrant parts of the first organized complete creatures, issuing directly from the hands of the Creator."

Nevertheless, the formal expression of the theory is, that the male semen provokes the development of a pre-existent germ, and that the ovaries of Eve, the first woman, contained the germs of all her immediate descendants, and *these* of all the members of the human family for ever to be born, and so for each and every animal this exists.

Harvey's studies led him to oppose the doctrine of pre-existences and to advocate warmly that of epigenesis, according to which, the being generated is created anew in all its parts, and this theory has been steadily gaining ground ever since his time, in spite of the defection of the great Haller, and of the opposition of such men as Bonnetus and Spallanzani.

"The theory of encasement," says Fred'k Cuvier, "sends back the difficulties of conception to a remote period, but does not resolve them." And Flourens, in our own time, sums up the arguments in favor of *epigenesis* in the following terms:

"I hold," says that philosopher, "that the male and female have each an equal share in the production of the germ; wherefore the study of the cross-breeds or métis, is the most profitable of all studies pursued in this connection. If the male jackall be made to cover a bitch, the progeny will be half jackall and half of the dog kind; if one of these half-breeds be impregnated by a male jackall, the offspring will be three-fourths jackall; if one of these be made to litter by a male jackall, the creatures born of this union will be almost entirely jackall; and so on until complete extinction of the dog. Now the germs of all these individuals were certainly not formed in the beginning, else I could not produce at pleasure, an animal of a certain kind, or extinguish, after a few generations, all traces of its distinguishing features. Besides, in the perfect working of nature, no monster can be supposed to have been created. They exist however, and their existence is, to a certain extent, an argument, nay, almost a positive proof against the doctrine of pre-existences."

I have but a word to say to you about equivocal generation, and here I quote Valentin. "The hypothesis of spontaneous generation was very favorably entertained," says the Professor of Berne, "in the infancy of science. It was then supposed that many of the lowest plants, and a large number of invertebrate animals, owed their immediate origin to putrefying organic substances; as did also some of the phanerogamous plants and many species of vertebrate animals. But a more careful study of the phenomenon of propagation, subsequently proved that that in all the higher beings the ordinary parental generation was the exclusive method; and that it even obtained in many lower animals, which were, perhaps also producible by spontaneous generation. And the farther the history of development was traced, the more the defenders of heterogeneous propagation were obliged to limit their hypothesis, so that, finally, the only instances in which it could be defended with any appearance of reason, were those of the *mould-plants*, confervae and infusoria, that appear in putrefying liquids; together with certain parasites, and the seminal corpuscles, which were then regarded as genuine animalcules."

A careful examination must withdraw even these last supports from the doctrine of non-parental generation.

"The more," observes Longet, a corroborative witness, "the question is fathomed, the more the hypothesis of spontaneous generation fades away."

"If spontaneous generation can be imagined, nothing positive justifies it; experience and observation, every day wiser and wiser, have snatched away one by one, the facts which constituted its strongest arguments; and those which the insufficiency of our knowledge still permits it to claim, are, to say the least, insignificant."

Day by day, gentlemen, are we gaining a newer and better acquaintance with the population of the sun beam, and the inhabitants of the micro-oceanic rain drop, with the succession of metamorphoses of animals in what is called "alternating generation," and the successive migrations of these animals as they become invested with each new form. We may wonder at the resurrection of dried animalcules, but we have learned not to confound it with spontaneous generation; and patient investigations have set aside the famous sulphuric acid experiments of Schultze.

I have not intended, gentlemen, to treat the whole subject of generation, but to consider it with reference to some particular comprehensive facts, which known to the inquisitive *mind* of the ancients, were made actual discoveries by the moderns; and also to some of the most popular theories which were, in the beginning, the offspring of prophetic vision, and in latter times were endowed with new animation by positive research. In the higher animals the human mind cannot possibly penetrate the mystery attending the union of ovule and spermatic fluid, for the understanding is limited to the observance and recognition of a wonderful series of facts, and is not permitted to enjoy a comprehension of *the essence* of the phenomena, for the comprehension of *essences* belongs to the Creator only. We may better know these facts, and be able to follow, moment by moment, every change of the initial matter until the completion of the perfect being—but the *wherefore*, will forever remain a mystery unfathomable by speculation.

